# Supplementary material for: Chemotherapy and Survival in Patients with Primary High-Grade Extremity and Trunk Soft Tissue Sarcoma
Source: Cancers (Basel). 2020 Aug 24;12(9):2389. doi: 10.3390/cancers12092389 (PMC7564235; doi:10.3390/cancers12092389)
Supplement: Supplementary file 1 [file cancers-12-02389-s001.zip › cancers-867102-supplementary-layout/Survival_Cancers_Supplementary_Figures.docx]

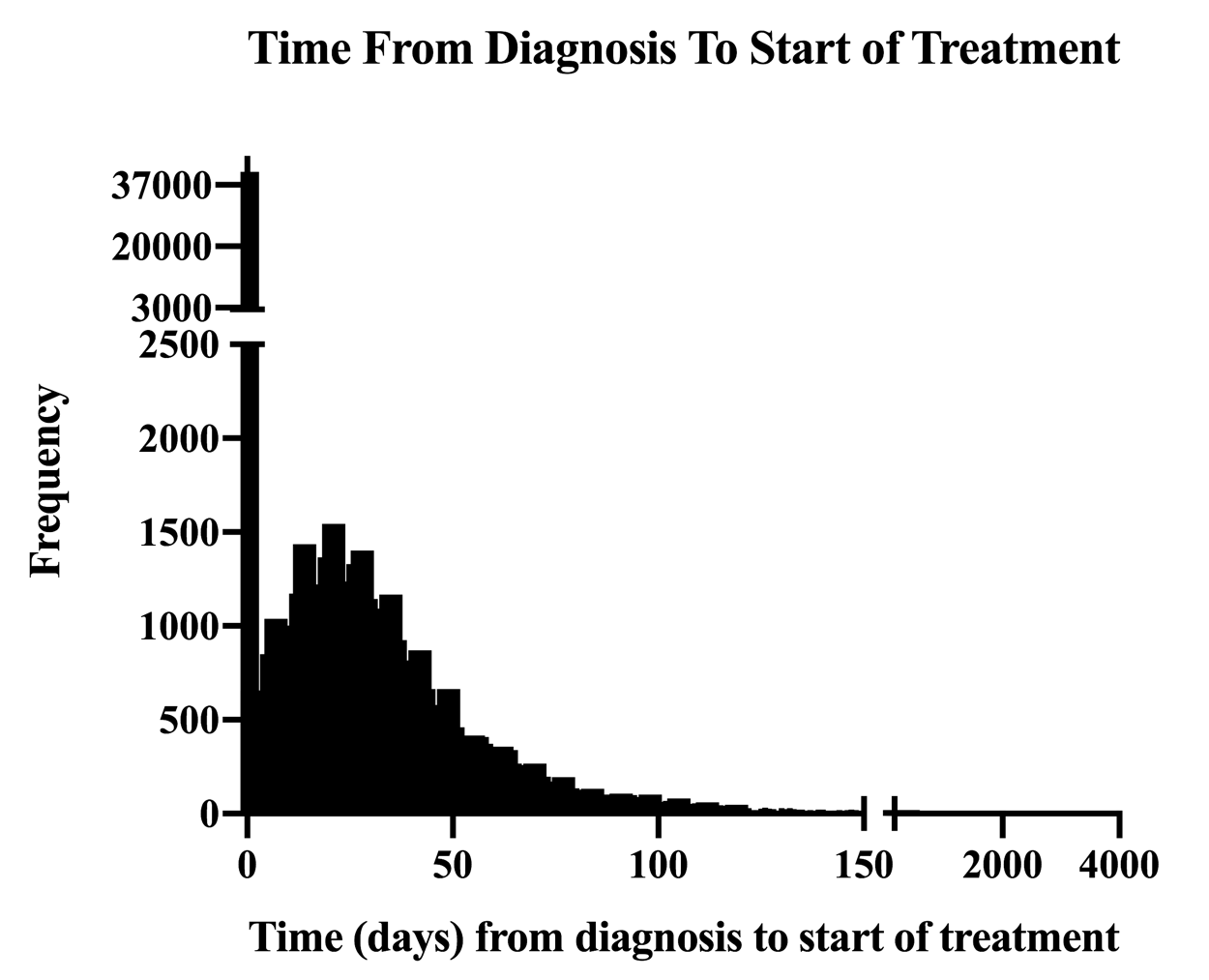


**Figure S1:** Histogram demonstrating the overall time from diagnosis to the start of treatment in the NCDB from 2004-2016. This calculation was repeated for multiple subgroups of patients, including those treated with any combination of surgery or neoadjuvant and/or adjuvant radiation (data not shown). CI = confidence interval; HR = hazard ratio; NCDB = National Cancer Database = NCDB; OS = overall survival; PSM = propensity score matching; STS = soft tissue sarcoma.

**Figure S2.** Analysis of annual sarcoma case volume (by facility) in the NCDB from 2004–2016. Facilities in the 99^th^ percentile performed >55 cases per year. One facility performed 160 cases per year, and the next highest volume facility performed 99 cases per year.

**Figure S3:** Distance from patient residence to treatment facility for adult patients with primary, high-grade, localized STS in the NCDB from 2004–2016.
